# Fasting Insulin and Risk of Overall and 14 Site-Specific Cancers: Evidence From Genetic Data

**DOI:** 10.3389/fonc.2022.863340

**Published:** 2022-04-21

**Authors:** Han Zhang, Doudou Li, Xiaozhuan Liu, Zhongxiao Wan, Zengli Yu, Yuming Wang, Xue Li

**Affiliations:** ^1^ Henan Provincial People’s Hospital, Zhengzhou University, Henan, China; ^2^ College of Public Health, Zhengzhou University, Henan, China

**Keywords:** fasting insulin, type 2 diabetes, cancer, Mendelian randomization study, polygenic risk score analysis

## Abstract

**Objective:**

Whether fasting insulin (FI) plays a role in cancer risk remains unclear. This study aimed to investigate the association between FI and cancer risk and to explore its potential mediator role in the association between type 2 diabetes mellitus (T2DM) and cancer.

**Methods:**

Two-sample Mendelian randomization (TSMR) analysis was performed to evaluate the effect of FI on overall and 14 site-specific cancers using genome-wide association study (GWAS) summary-level data from Meta-Analyses of Glucose and Insulin-related traits Consortium (MAGIC) and consortia of 14 site-specific cancers. The primary MR approach was conducted by using the random-effect inverse-variance weighted (IVW) method, and sensitivity analyses were implemented by adopting weighted-median, weighted-mode, MR-Egger, and MR-PRESSO tests. Polygenic risk score analysis was executed by using individual-level data from UK Biobank to validate the findings from TSMR analyses. Multivariable Mendelian randomization (MVMR) was carried out to estimate the mediation effect of FI on the association between T2DM and cancer.

**Results:**

TSMR study suggested that genetically determined high FI levels were associated with increased risk of colorectal cancer (odds ratio (OR) = 1.87, 95% CI: 1.23–2.84, *p* = 0.003) and endometrial cancer (OR = 1.89, 95% CI: 1.08–3.01, *p* = 0.008), but not associated with overall cancer risk or the other 12 studied cancer sites. Polygenic risk score analysis successfully replicated the association between genetic liability to high FI levels and the increased risk of colorectal and endometrial cancers. MVMR and MR mediation analyses detected an intermediary effect of FI and quantified that FI mediated 21.3% of the association between T2DM and endometrial cancer.

**Conclusions:**

This study demonstrated that FI levels are associated with the risk of colorectal and endometrial cancers, and FI was found to play an intermediary role in the association between T2DM and endometrial cancer. The associations between FI and other cancers need to be further studied.

## Introduction

Fasting insulin (FI), a peptide hormone, plays an indispensable role in regulating blood glucose and energy metabolism. Evidence from observational studies has indicated that elevated FI levels may be related to increased risk of overall cancer (1) and site-specific cancers, such as breast (2), colorectal (3), prostate (4, 5), pancreatic (6, 7), kidney (8), uterine (9), gastric (10), lung (11), and liver cancers (12). Besides, a comprehensive meta-analysis of observational studies has demonstrated that type 2 diabetes mellitus (T2DM) was associated with developing several cancers (13), including hepatocellular, hepatobiliary, pancreas, breast, ovarian, endometrial, and gastrointestinal cancers. However, because of the possible residual confounding and reverse association of these observational studies, the effects of FI and T2DM on cancer risk are not fully established. Furthermore, although several studies have reported possible mechanisms underlying diabetes and cancer, such as hyperglycemia (14), hyperinsulinemia (15), and increased activity of insulin-like growth factor 1(IGF-1) (16), the role of FI in the association between T2DM and cancer remains unclear. Considering that FI levels can be modified by diet control (17, 18) and medical therapy (19), establishing the association between FI and cancer risk and exploring its effect on the association between T2DM and associated cancer are crucial from clinical and public health perspectives.

Compared with conventional observational studies, Mendelian randomization (MR) study, by exploiting genetic variants (usually single-nucleotide polymorphisms (SNPs)) as instrumental variables (IVs) of the exposure, can enhance the exposure–outcome inference by diminishing possible confounding and avoiding the reverse correlation (20, 21). This method minimizes confounding because genetic variants are randomly assorted at conception and therefore have nothing to do with self-adapted lifestyle and environmental factors. Since allelic randomization precedes the occurrence of diseases, the MR framework can also overcome inverse association. Recently, this method has been extended to multivariable MR (MVMR) (22, 23), which can be used to evaluate the independent direct effects of multiple and potentially related exposures, as well as to estimate the proportion of the exposure–outcome effect mediated by a mediator or set of mediators when combined with MR (24).

Given the inconclusive evidence and natural methodological limitations of previous observational studies on FI levels and cancer risk, the primary objective of this study is to determine the association between FI levels and the risk of overall and 14 site-specific cancers by conducting two-sample MR (TSMR) framework and polygenic risk score (PRS) analyses. Furthermore, we performed MVMR and MR mediation analyses to explore the mediation effect of FI on the association between T2DM and cancer risk.

## Methods

### Study Design

**Figure 1:**
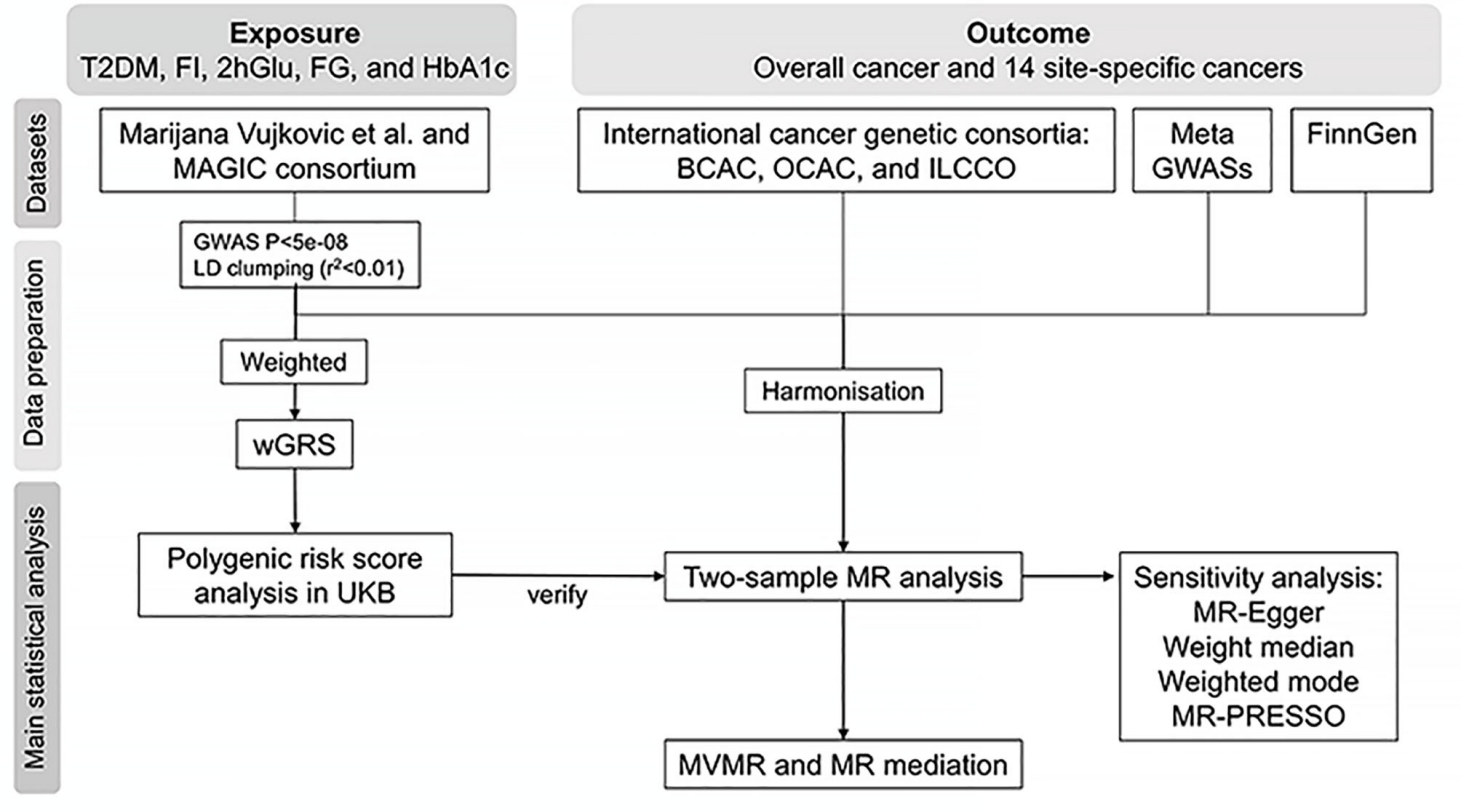
Flowchart of the study. T2DM, type 2 diabetes; FI, fasting insulin; 2hGlu, 2-h glucose after an oral glucose challenge; FG, fasting glucose; HbA1c, glycated hemoglobin; LD, linkage disequilibrium; wGRS, weighted genetic risk score; UKB, UK Biobank; BCAC, the Breast Cancer Association Consortium; OCAC, the Ovarian Cancer Association Consortium; ILCCO, the International Lung Cancer Consortium; MR, Mendelian randomization; MVMR, multivariable MR.

To assess the associations between FI and the risk of overall cancer and 14 site-specific cancers as well as the possibly intermediary effect of FI on the association between T2DM and cancer, a TSMR framework was first performed based on genome-wide association study (GWAS) summary-level data from several consortia for overall cancer and site-specific cancers. Then PRS analysis using individual-level data from UK Biobank was conducted to further validate the associations between genetically determined FI levels and cancer risk. Finally, the MVMR and mediation analyses were carried out to explore the potential intermediary effect of FI on the association between T2DM and cancer. Individual-level UK Biobank data were accessed under the approved project application (ID: 66354). The study design is presented in **Figure 1**.

### Genetic Instruments of Glycemic Traits and Type 2 Diabetes Mellitus

Single-nucleotide polymorphisms (SNPs) for glycemic traits [including FI, fasting glucose (FG), 2-h glucose after an oral glucose challenge (2hGlu), and glycated hemoglobin (HbA1c)] were derived from the Meta-Analyses of Glucose and Insulin-related traits Consortium (MAGIC) including 281,146 participants (25). For T2DM, the genetic instruments were extracted from a multi-ethnic meta-analysis among 1.4 million participants (26). For the SNPs with a genome-wide significance among those of European descent (*p* < 5×10^−8^), linkage disequilibrium (LD) was evaluated among these SNPs, and the F-statistic for each of them was calculated. Genetic instruments with a low LD level (r^2^ < 0.01) and high F-statistics (F > 10) were finally included in the MR analysis. Briefly, a total of 380, 36, 69, 14, and 50 SNPs were selected as IVs for T2DM, FI, FG, 2hGlu, and HbA1c, respectively. Trait variance explained by genetic IVs for T2DM, FI, FG, 2hGlu, and HbA1c was 35.5%, 0.8%, 3.9%, 0.9%, and 5.2% (25, 26), respectively. The details of the used SNPs are displayed in **Supplementary Table 1**.

### Data Sources for Overall Cancer and Site-Specific Cancers

Summary-level genetic data of overall cancer and 14 site-specific cancers were taken from the FinnGen Biobank, the Breast Cancer Association Consortium (BCAC), the UKB and the Kaiser Permanente Genetic Epidemiology Research on Adult Health and Aging (GERA) cohorts, the Ovarian Cancer Association Consortium (OCAC), the International Lung Cancer Consortium (ILCCO), a meta-analysis of 13 studies of endometrial cancer and the Epidemiology of Endometrial Cancer Consortium (E2C2), and a meta-analysis of 11 previous colorectal cancer (CRC) GWASs (**Supplementary Table 2**). To enhance the statistical power, only cancer types with more than 400 cases were included in the TSMR analysis. The details of the outcome data source are described in the **Supplementary Material**.

### Two-Sample Mendelian Randomization Analysis and Sensitivity Analysis

The inverse-variance weighted (IVW) approach was adopted as the primary analysis to determine the associations between FI levels and different types of cancer. Model-based estimates (27), weighted median (WM) method (28), and MR pleiotropy residual sum and outlier (MR-PRESSO) test (29) were also performed to further assess the robustness of the associations. Cochrane’s *Q* statistic was calculated to measure the heterogeneity, and the MR-Egger test (intercept *p* ≤ 0.05) was conducted to detect possible pleiotropy. If more than 50% of the weight comes from valid instruments, the weighted-median method provides consistent estimates of associations even when horizontal pleiotropy exists (30). Since the MR-PRESSO test can detect possible outliers and provide estimates after the removal of outliers, it was implemented to correct for possible pleiotropic effects (29). In addition, scatter plots were employed to compare MR models, and “leave-one-out” analyses were also used to detect outliers for SNPs (31). The “TwoSampleMR” R package and “MR-PRESSO” package were applied for these analyses in R4.1.2 software. All statistical analyses were two-tailed.

### Polygenic Risk Score Analysis in UK Biobank

PRS analysis was conducted by using individual-level data from the UK Biobank (32). The cancer cases were defined by corresponding codes in International Classification of Diseases (ICD) versions 9 and 10 with information from national medical records, which included inpatient hospital episode records, cancer registry, and death registry. The PRSs were constructed by summing up the number of risk-increasing alleles for each SNP weighted by their effect size (beta) on FI levels and then adding this weighted score for all used SNPs. The details of the used SNPs are displayed in **Supplementary Table 3**. Multivariate logistic regression was performed subsequently to explore the association between the PRS of FI and cancer risk with adjustment for age, sex, BMI, drinking, smoking, and the first 10 genetic principal components. Bonferroni correction was applied to correct the threshold of statistical significance for multiple comparisons, and the *p*-value below 0.0036 (where *p* = 0.05/14) in PRS analysis was considered to be strong evidence of significant association, while associations with a *p*-value below 0.05 were considered as suggestive significance. The PRS analysis was conducted by using R4.1.2 software.

### Multivariable Mendelian Randomization and Mediation Analysis

Given the overlap in the genetic variables of T2DM with FI, whether FI may play an intermediary role in the association between T2DM and a certain type of cancer was assessed by using the MVMR and MR mediation analyses. In this stage, MVMR analysis was applied in a two-sample setting to adjust for the genetic association of the instruments with FI. Subsequently, network MR analysis was carried out to estimate the proportion of the effect of T2DM on certain cancer mediated by FI (33). Analyses at this stage were also implemented through the “TwoSampleMR” package in R4.1.2 software.

## Results

### Two-Sample Mendelian Randomization Analysis

**Figure 2:**
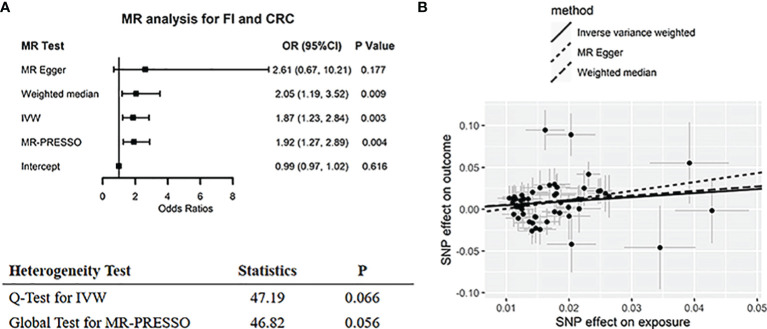
Two-sample MR analysis results of FI on colorectal cancer. **(A)** Forest plot for summarizing the results of all MR methods. The black box represents the effect estimates, and the error line represents the 95% CI. **(B)** Scatter plot for comparison of MR methods. The black circle represents the point effect estimate, and the slope represents the correlation trend. FI, fasting insulin; CRC, colorectal cancer; MR, Mendelian randomization; IVW, inverse-variance weighted; MR-PRESSO, MR pleiotropy residual sum and outlier test; OR, odds ratio.

**Figure 3:**
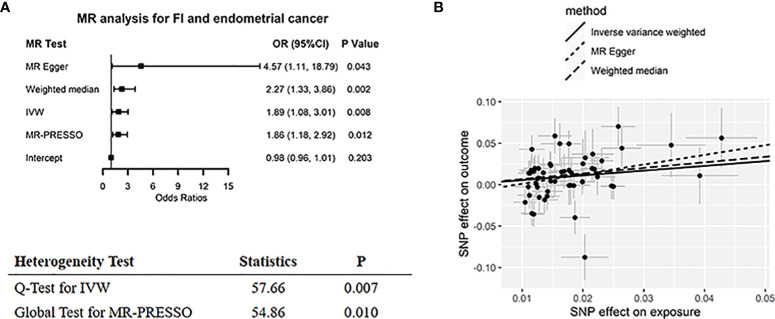
Two-sample MR analysis results of FI on endometrial cancer. **(A)** Forest plot for summarizing the results of all MR methods. The black box represents the effect estimates, and the error line represents the 95% CI. **(B)** Scatter plot for comparison of MR methods. The black circle represents the point effect estimate, and the slope represents the correlation trend. FI, fasting insulin; CRC, colorectal cancer; MR, Mendelian randomization; IVW, inverse-variance weighted; MR-PRESSO, MR pleiotropy residual sum and outlier test; OR, odds ratio.

MR IVW approach suggested that genetically determined high FI levels were associated with increased risk of colorectal (odds ratio (OR) = 1.87, 95% CI: 1.23–2.84, *p* = 0.003) (**Figure 2A**) and endometrial cancer (OR = 1.89, 95% CI: 1.08–3.01, *p* = 0.008) (**Figure 3A**). Sensitivity analysis comprising MR-Egger, WM, and MR-PRESSO showed a similar association pattern (all OR > 1). The *p*-values of MR-Egger interception for colorectal and endometrial cancers were 0.616 and 0.203, respectively, indicating there was no horizontal pleiotropy. Although outlier (n = 1) was detected in the MR-PRESSO test for endometrial cancer, the effect estimate (*β* = 0.71, *p* = 0.002) with the removal of outliers was similar to the effect estimate (*β* = 0.62, *p* = 0.012) without removal (*p*-value of distortion test > 0.05, as shown in **Supplementary Table 4**). Scatter plots illustrated consistent estimate trends among three MR methods (**Figures 2B** and **3B**), indicating the robustness of these results. The “leave-one-out” MR analysis also reported consistent significant associations between FI level and the risk of colorectal and endometrial cancers (**Supplementary Figure 1**).

However, there was no evidence supporting an association of FI with other cancers and overall cancer (all *p*_effect_ > 0.05) (**Supplementary Table 5**). The results of sensitivity analyses for FI and other cancers showed consistent estimates (**Supplementary Table 3** and **Supplementary Figure 1**).

**Table 1** depicts the TSMR results of other glycemic traits and T2DM on colorectal and endometrial cancers. There was no association of other glycemic traits with colorectal cancer (all *p*_effect_ > 0.05), and the sensitivity analyses presented consistently null estimates (all *p*_effect_ > 0.05) (**Supplementary Figure 2**). However, we found a significant association between genetic liability to T2DM and increased endometrial cancer risk (OR = 1.10, 95% CI: 1.04–1.16, *p* = 0.001). Although the Q-test of the IVW approach (*p*_heterogeneity_ < 0.001) showed obvious heterogeneity, the sensitivity analyses illustrated consistent results with the primary analysis (**Supplementary Figure 3**), and there was no horizontal pleiotropy (*p*_intercept_ = 0.097).

**Table 1:** Two-sample MR analysis results of glycemic traits and T2DM on colorectal and endometrial cancers.

Cancer type	Exposure	N of SNPs	Methods	OR (95% CI)	*p*_effect_	*p*_heterogeneity_	*p*_intercept_^†^
CRC			MR-Egger	2.61 (0.67, 10.21)	0.177	0.056	0.616
			Weighted median	2.05 (1.19, 3.52)	0.009		
	FI	35	IVW	1.87 (1.23, 2.84)	0.003^*^	0.066	
			Simple mode	2.52 (0.91, 6.98)	0.084		
			Weighted mode	2.18 (0.89, 5.33)	0.098		
			MR-Egger	1.22 (0.74, 1.99)	0.438	0.001^#^	0.716
			Weighted median	1.04 (0.76, 1.72)	0.802		
	FG	58	IVW	1.13 (0.87, 1.45)	0.365	0.001^#^	
			Simple mode	1.02 (0.50, 2.06)	0.959		
			Weighted mode	1.04 (0.77, 1.41)	0.800		
			MR-Egger	0.53 (0.19, 1.42)	0.210	0.001^#^	0.126
			Weighted median	1.02 (0.65, 1.62)	0.921		
	HbA1c	45	IVW	1.07 (0.70, 1.65)	0.753	<0.001^#^	
			Simple mode	0.95 (0.43, 2.11)	0.900		
			Weighted mode	1.02 (0.56, 1.84)	0.953		
			MR-Egger	0.91 (0.52, 1.59)	0.750	0.079	0.964
			Weighted median	0.96 (0.78, 1.18)	0.685		
	2hGlu	12	IVW	0.90 (0.75, 1.07)	0.239	0.113	
			Simple mode	1.04 (0.74, 1.47)	0.821		
			Weighted mode	1.01 (0.78, 1.30)	0.944		
			MR-Egger	0.93 (0.83, 1.05)	0.238	<0.001^#^	0.067
			Weighted median	1.01 (0.94, 1.09)	0.740		
	T2DM	326	IVW	1.03 (0.98, 1.08)	0.227	<0.001^#^	
			Simple mode	1.08 (0.87, 1.36)	0.483		
			Weighted mode	1.03 (0.86, 1.22)	0.756		
EC			MR-Egger	4.57 (1.11, 18.79)	0.043	0.010^#^	0.203
			Weighted median	2.27 (1.33, 3.86)	0.002		
	FI	35	IVW	1.89 (1.08, 3.01)	0.008*	0.007^#^	
			Simple mode	2.86 (1.07, 7.66)	0.044		
			Weighted mode	2.46 (1.00, 6.02)	0.057		
			MR-Egger	1.24 (0.72, 2.12)	0.441	<0.001^#^	0.404
			Weighted median	1.04 (0.74, 1.46)	0.836		
	FG	59	IVW	1.02 (0.77, 1.34)	0.913	<0.001^#^	
			Simple mode	1.22 (0.58, 2.56)	0.594		
			Weighted mode	1.20 (0.85, 1.69)	0.314		
			MR-Egger	0.96 (0.43, 2.19)	0.931	<0.001^#^	0.991
			Weighted median	1.26 (0.81, 1.95)	0.303		
	HbA1c	46	IVW	0.96 (0.62, 1.49)	0.856	<0.001^#^	
			Simple mode	1.05 (0.51, 2.19)	0.891		
			Weighted mode	1.18 (0.78, 1.79)	0.441		
			MR-Egger	0.60 (0.29, 1.22)	0.188	0.002^#^	0.226
			Weighted median	0.88 (0.70, 1.10)	0.261		
	2hGlu	12	IVW	0.93 (0.72, 1.20)	0.568	0.001^#^	
			Simple mode	0.89 (0.61, 1.30)	0.553		
			Weighted mode	0.86 (0.61, 1.20)	0.389		
			MR-Egger	0.99 (0.87, 1.13)	0.919	<0.001^#^	0.097
			Weighted median	1.05 (0.98, 1.13)	0.203		
	T2DM	328	IVW	1.10 (1.04, 1.16)	0.001*	<0.001^#^	
			Simple mode	1.04 (0.84, 1.29)	0.697		
			Weighted mode	1.00 (0.88, 1.14)	0.994		

CRC, colorectal cancer; EC, endometrial cancer; T2DM, type 2 diabetes; FI, fasting insulin; 2hGlu, 2-h glucose after an oral glucose challenge; FG, fasting glucose; HbA1c, glycated hemoglobin; OR, odds ratio; IVW, inverse-variance weighted; MR, Mendelian randomization; SNPs, single-nucleotide polymorphisms.

^*^p_effect_ less than 0.05, which represents statistical significance.

^#^p_heterogeneity_ less than 0.05, which indicates that there is heterogeneity across the used SNPs.

**^†^** p_intercept_ more than 0.05, which indicates that there is no pleiotropy of the used SNPs.

### Polygenic Risk Score Analysis in UK Biobank

**Figure 4:**
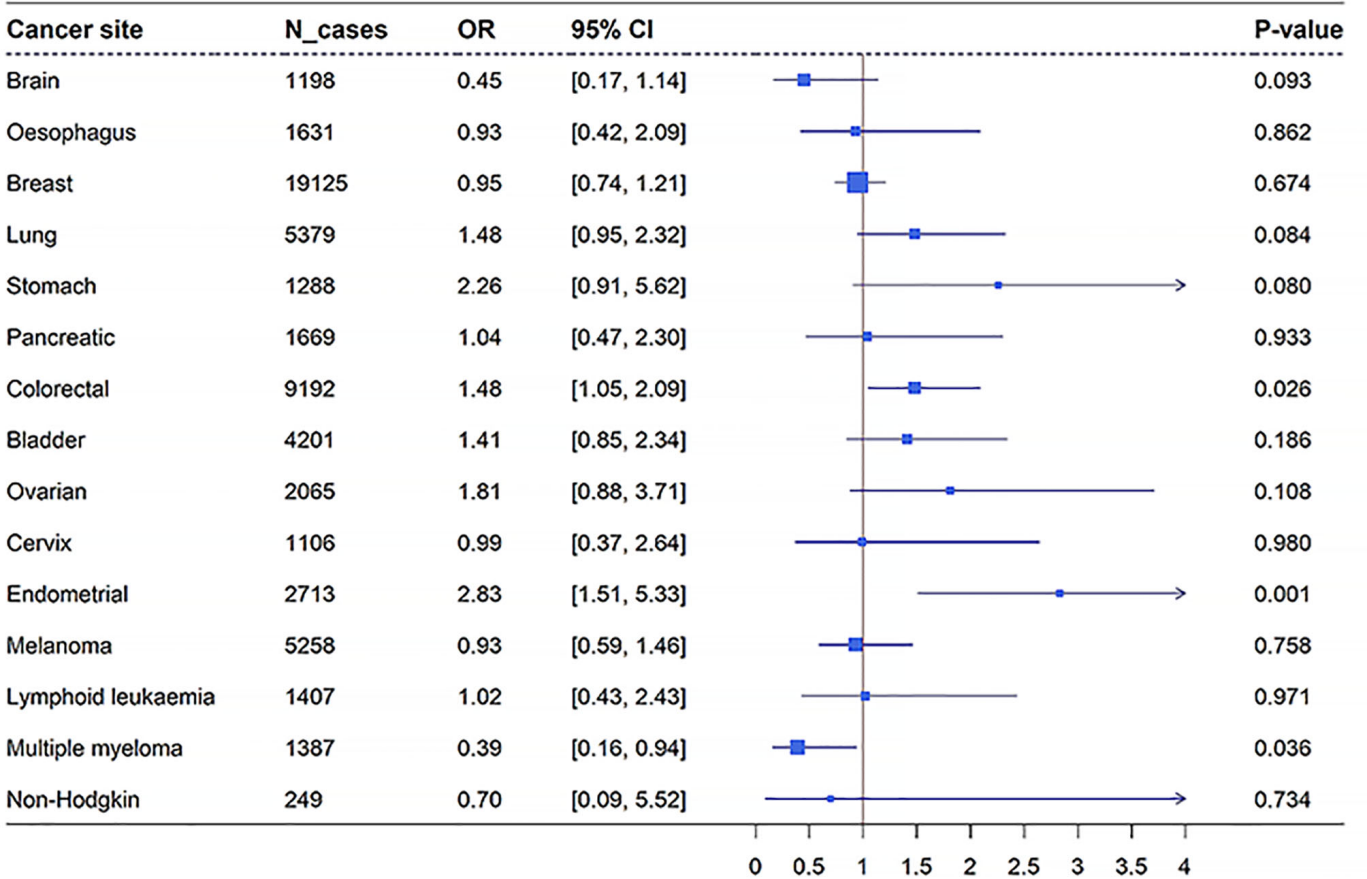
Forest plot for the results of PRS analysis of FI on overall cancer and site-specific cancers in UK Biobank. The blue box represents the effect estimates, and the blue error line represents the 95% CI. OR, odds ratio; PRS, polygenic risk score; FI, fasting insulin.

With adjustment for age, sex, BMI, drinking, smoking, and the first 10 genetic principal components in a multivariable logistic regression model, a significant association was observed between genetically determined high levels of FI and the increased risk of endometrial cancer (OR = 2.83, 95% CI: 1.51–5.33, *p* = 0.001) (**Figure 4**). There was suggestive evidence supporting the association between genetically determined high FI levels and increased risk of colorectal cancer (OR = 1.48, 95% CI: 1.05–2.09, *p* = 0.026). As displayed in **Figure 4**, we did not find any significant association between genetically related FI levels with other site-specific cancers (all *p* > 0.05).

### Multivariable Mendelian Randomization and Mediation Analysis

**Figure 5:**
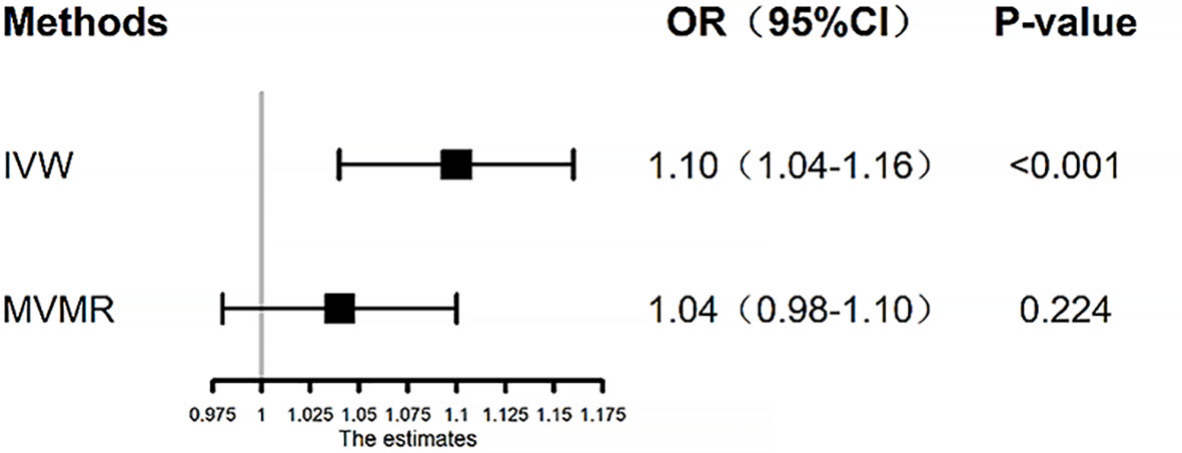
Inverse-variance weighted (IVW) and multivariable Mendelian randomization (MVMR) estimates for genetic liability to T2DM on risk of endometrial cancer. The MVMR analysis adjusts for the associations of the genetic instruments with fasting insulin. The black box represents the effect estimates, and the black error line represents the 95% CI. IVW, inverse-variance weighted; OR, odds ratio; T2DM, type 2 diabetes mellitus.

Based on the results from TSMR, we performed MVMR analysis to adjust for genetically determined FI and T2DM in the same model. We found that the effect estimates of T2DM on endometrial cancer (OR = 1.04, 95% CI: 0.98–1.10, *p* = 0.224) were obviously attenuated when compared with the primary univariable MR analysis (OR = 1.10, 95% CI: 1.04–1.16, *p* < 0.001) (**Figure 5**). The residual MVMR method illustrated similar results (**Supplementary Table 6**). The results of network MR analysis are presented in **Supplementary Table 7**; the ORs of T2DM and FI on endometrial cancer were 1.04 (95% CI: 0.98–1.10, *p* = 0.224) and 2.75 (95% CI: 1.60–4.73, *p* < 0.001), respectively. Moreover, increased risk of T2DM was also associated with increased FI levels (OR = 1.02, 95% CI: 1.01–1.03, *p* = 0.001). These results indicated that the effect of T2DM on endometrial cancer may be mediated through FI. Subsequently, we performed MR mediation analysis and quantified the intermediary effect of FI on the association between T2DM and endometrial cancer as 21.3%.

## Discussion

In this study, we found strong evidence of positive associations between FI levels and the risk of colorectal and endometrial cancers. Furthermore, MVMR and MR mediation analyses demonstrated that FI plays an intermediary role in the association between T2DM and endometrial cancer.

Several observational studies have found that higher FI levels were related to increased colorectal cancer risk (34–36). Consistent with our finding, a case–control study reported that fasting blood insulin levels were higher in non-diabetic colorectal cancer patients compared with obesity-matched controls and that FI levels were associated with increased colorectal cancer risk (34). Similarly, a case–control study from Korea with 3,606 cases and 6,019 controls indicated that increased serum insulin levels and insulin insistence were significantly associated with the presence of colorectal cancer (36). A meta-analysis of 35 studies involving 25,566 cases showed that higher FI levels were significantly associated with an increased risk of colorectal cancer (37). A recent MR study also validated the association between FI and colorectal cancer risk (38). In contrast, a longitudinal study with a median follow-up period of 11.9 years demonstrated that serum insulin was not related to the risk of colorectal cancer in postmenopausal women (39). Furthermore, a previous MR study found no evidence supporting the association between FI levels and colorectal cancer risk (8). The discrepancy may be due to the small number of genetic instruments used and the small proportion of variance explained by IVs in the previous MR study.

The results of the current study are consistent with previous findings supporting a harmful role of high FI levels in endometrial cancer. Increased serum insulin has been reported to be associated with roughly a doubling of endometrial cancer risk in postmenopausal women (40). A systematic review and meta-analysis (13 studies, n = 4,088) provided suggestive evidence that high FI was related to an increased risk of endometrial cancer (41). An MR study also reported a positive association between genetically determined higher FI levels and greater risk of endometrial cancer (42). In addition, observational and meta-analysis studies have illustrated a positive association between T2DM and endometrial cancer (43–45). An umbrella review of observational and MR studies also identified potential associations for genetic predisposition to T2DM and FI concentrations and risk of endometrial cancer (46). However, the intermediary role of FI in the association between T2DM and endometrial cancer was not reported. Our study observed that FI mediated around 23.1% of the effect of T2DM on endometrial cancer.

In line with our findings, a TSMR study found limited evidence of the association of genetically determined FI levels with overall cancer (8). However, a prospective cohort study showed higher cancer mortality in patients with hyperinsulinemia regardless of obesity (47). Another observational study also has proposed that extremely high FI levels were probably an independent risk of cancer mortality among men rather than women (48). Since the present study mainly focused on the overall cancer risk, we could not conclude that the results of this work were inconsistent with those observational studies.

The advantage of our study is the MR study design, which can diminish confounding and reverse correlation, potentially biasing the findings of observational studies. Secondly, considering that many metabolism-related traits may affect each other, we also performed MVMR and MR mediation analyses to rule out the possible influence and to explore the potential effect of FI on the association between T2DM and endometrial cancer. To our knowledge, the IVs for glycemic traits used in this study are the most up to date and comprehensive, and it can enhance the statistical power of this work. We performed the analysis only among European populations, which largely avoided bias caused by population stratification. However, it also confined the transferability of our results to other populations at the same time. Another major limitation of the study is that the number of cases was few for certain site-specific cancers, which would reduce precisely the estimates. Additionally, given the relatively small proportion of variance explained by FI-related genetic variants, our study might lack the power to identify weak or moderate associations. It should be acknowledged that although MR design is advantageous in the association inference, the claim of causality should be taken with caution given the complexity of association and the limitations of the MR study. In order to increase the reliability of the results, we conducted PRS analysis in UK Biobank to validate the findings of the TSMR study. However, given that UK Biobank did not have data on circulating FI levels, we were therefore not able to estimate the variance of FI levels explained by the PRS. In the future, larger observational and well-designed experimental studies are warranted to explore the complex role and mechanism of FI in cancer progression.

In summary, this MR study provided strong evidence of positive associations of FI with colorectal and endometrial cancer risk. Our study also demonstrated an intermediary effect of FI on the association between T2DM and endometrial cancer. However, there was limited evidence in support of associations between FI and overall cancer and other site-specific cancers. In addition to the benefits of insulin for diabetes treatment, it is suggested that regular monitoring of insulin levels and screening and treatment for subclinical insulinemia may be an efficient colorectal and endometrial cancer prevention strategy.

## Data Availability Statement

The original contributions presented in the study are included in the article/**Supplementary Material**. Further inquiries can be directed to the corresponding authors.

## Author Contributions

Conceptualization: XL and YW. Methodology: XL. Software: HZ. Validation: HZ, DL, and XZL. Formal analysis: HZ. Investigation: DL and XZL. Resources: XZL. Data curation: ZY and YW. Writing—original draft preparation: HZ. Writing—review and editing: ZW, XL, YW, and ZY. Visualization: HZ. Supervision: XL and YW. Project administration: XL. All authors listed have made a substantial, direct, and intellectual contribution to the work and approved it for publication.

## Conflict of Interest

The authors declare that the research was conducted in the absence of any commercial or financial relationships that could be construed as a potential conflict of interest.

## Publisher’s Note

All claims expressed in this article are solely those of the authors and do not necessarily represent those of their affiliated organizations, or those of the publisher, the editors and the reviewers. Any product that may be evaluated in this article, or claim that may be made by its manufacturer, is not guaranteed or endorsed by the publisher.
