# Supplementary material for: Fasting Insulin and Risk of Overall and 14 Site-Specific Cancers: Evidence From Genetic Data
Source: Front Oncol. 2022 Apr 21;12:863340. doi: 10.3389/fonc.2022.863340 (PMC9069016; doi:10.3389/fonc.2022.863340)
Supplement: Supplementary file 1 [file DataSheet_1.docx]

**Title: Fasting insulin and risk of overall and 14 site-specific cancers：evidence from genetic data**

**Supplementary Materials of methods**

The details of each outcome data source were as follows:

Summary-level genetic data for overall cancer, lymphoid leukaemia, multiple myeloma and malignant plasma cell neoplasms, and brain cancer came from FinnGen consortium, and were based on 218,792 European-descent participants (38,036 cancer cases and 180,756 control subjects), 218,792 European-descent participants (663 lymphoid leukaemia cases and 218,129 control subjects), 218,792 European-descent participants (598 multiple myeloma and malignant plasma cell neoplasms cases and 218,194 control subjects), 218,792 European-descent participants (464 brain cancer cases and 218,328 control subjects), respectively. The detailed contents about FinnGen consortium, including participating biobanks/cohorts, data collection, genotyping and data analysis, are presented on its webpage ^[1]^.

Summary-level genetic data for breast cancer came from the BCAC consortium ^[2]^, and were based on 228,951 European-descent participants (122,977 breast cancer case and 105,974 control subjects). The GWAS based on the BCAC used 1000 Genomes Project (Phase 3) reference panel in imputation stage and adjusted for genetic principal components and country.

Summary-level genetic data for cervix cancer, pancreatic cancer, bladder cancer, non-Hodgkin lymphoma, melanoma, and Esophagus/stomach cancer came from the UKB ^[3]^ and the GERA cohorts ^[4, 5]^, and were based on 416,913 European ancestry participants (6563 cervix cancer case and 410,350 control subjects), 411,013 European ancestry participants (663 pancreatic cancer case and 410,350 control subjects), 412,592 European ancestry participants (2042 bladder cancer case and 410,350 control subjects), 412,750 European ancestry participants (2400 non-Hodgkin lymphoma case and 410,350 control subjects), 417,127 European ancestry participants (6777 melanoma case and 410,350 control subjects), and 411,441 European ancestry participants (1091 esophagus/ stomach cancer case and 410,350 control subjects), respectively. The detailed methods were described by Sara R. Rashkin and colleagues ^[6]^.

Summary-level genetic data for ovarian cancer came from the OCAC consortium, and were based on 66,450 European ancestry participants (25,509 ovarian cancer case and 40,941 control subjects). Genotypes for OCAC samples were selected preferentially from OncoArray, MayoGWAS, the Collaborative Oncological Gene-environment Study (COGS) and other GWAS in order ^[7]^. The processes of data analysis and quality control were described by Catherine M. Phelan and colleagues ^[7]^.

Summary-level genetic data for lung cancer came from the ILCCA consortium ^[8]^, and were based on 27,209 European ancestry participants (11,348 ovarian cancer case and 15,861 control subjects). The data of ILCCA for all scans was imputed using data from the 1000 Genomes Project (Phase 1 integrated release 3, March 2012) ^[8]^.

Summary-level genetic data for endometrial cancer (EC) came from a meta-GWAS study including 17 studies identified via the Endometrial Cancer Association Consortium (ECAC), the E2C2 consortium and the UK Biobank, and were based on 121,885 European ancestry participants (12,906 EC case and 108,979 control subjects). The details of the data sources were published elsewhere ^[9]^.

Summary-level genetic data for colorectal cancer (CRC) came from our published meta-analysis of 11 previous CRC GWASs ^[10]^, and were based on 42,710 European ancestry participants (20,049 CRC case and 22,661 control subjects).

**References:**

[1] Yuan S, Larsson SC. An atlas on risk factors for type 2 diabetes: a wide-angled Mendelian randomisation study[J]. Diabetologia, 2020, 63(11): 2359-2371

[2] Michailidou K, Lindström S, Dennis J, et al. Association analysis identifies 65 new breast cancer risk loci[J]. Nature, 2017, 551(7678): 92-94

[3] Bycroft C, Freeman C, Petkova D, et al. The UK Biobank resource with deep phenotyping and genomic data[J]. Nature, 2018, 562(7726): 203-209

[4] Banda Y, Kvale MN, Hoffmann TJ, et al. Characterizing Race/Ethnicity and Genetic Ancestry for 100,000 Subjects in the Genetic Epidemiology Research on Adult Health and Aging (GERA) Cohort[J]. Genetics, 2015, 200(4): 1285-95

[5] Kvale MN, Hesselson S, Hoffmann TJ, et al. Genotyping Informatics and Quality Control for 100,000 Subjects in the Genetic Epidemiology Research on Adult Health and Aging (GERA) Cohort[J]. Genetics, 2015, 200(4): 1051-60

[6] Rashkin SR, Graff RE, Kachuri L, et al. Pan-cancer study detects genetic risk variants and shared genetic basis in two large cohorts[J]. Nat Commun, 2020, 11(1): 4423

[7] Phelan CM, Kuchenbaecker KB, Tyrer JP, et al. Identification of 12 new susceptibility loci for different histotypes of epithelial ovarian cancer[J]. Nat Genet, 2017, 49(5): 680-691

[8] Wang Y, McKay JD, Rafnar T, et al. Corrigendum: Rare variants of large effect in BRCA2 and CHEK2 affect risk of lung cancer[J]. Nat Genet, 2017, 49(4): 651

[9] O'Mara TA, Glubb DM, Amant F, et al. Identification of nine new susceptibility loci for endometrial cancer[J]. Nat Commun, 2018, 9(1): 3166

[10] Li X, Timofeeva M, Spiliopoulou A, et al. Prediction of colorectal cancer risk based on profiling with common genetic variants[J]. Int J Cancer, 2020, 147(12): 3431-3437
